# Draft Genome Sequence of *Aliivibrio fischeri* Strain 5LC, a Bacterium Retrieved from Gilthead Sea Bream (*Sparus aurata*) Larvae Reared in Aquaculture

**DOI:** 10.1128/genomeA.00593-15

**Published:** 2015-06-04

**Authors:** Gianmaria Califano, Telma Franco, Ana C. S. Gonçalves, Sara Castanho, Florbela Soares, Laura Ribeiro, Leonardo Mata, Rodrigo Costa

**Affiliations:** a Microbial Ecology and Evolution Research Group, Centre of Marine Sciences (CCMar), University of Algarve (UAlg), Gambelas, Faro, Portugal; b Department of Biological Sciences, State University of Santa Cruz, UESC, Ilhéus, Bahia, Brazil; c Portuguese Institute for the Sea and Atmosphere (IPMA), Aquaculture Research Station, Olhão, Portugal; d MACRO–the Centre for Macroalgal Resources and Biotechnology, James Cook University, Townsville City, Queensland, Australia

## Abstract

To shed light on the putative host-mediated lifestyle of the quintessential marine symbiont *Aliivibrio fischeri*, and on the symbiosis versus potentially pathogenic features of bacteria associated with farmed fish, we report the draft genome sequence of *A. fischeri* strain 5LC, a bacterium retrieved from gilthead sea bream (*Sparus aurata*) larvae.

## GENOME ANNOUNCEMENT

The bacterium *Aliivibrio fischeri*, formerly *Vibrio fischeri* (1), is widely known for its unique symbiotic relationship with the bobtail squid *Euprymna scolopes*, whereby the luminescent symbiont is thought to provide the host with sophisticated antipredatory skills through a highly specialized bipartite interaction (2). Recently, strains of *A. fischeri* have been associated with disease outbreaks in farmed fish (3), raising concerns about the pathogenic potential of *A. fischeri* in fish-rearing systems. To improve the understanding of the adaptive strategies of this species, we announce the draft genome sequence of *Aliivibrio fischeri* 5LC, a strain isolated from larvae of *Sparus aurata* (gilthead seabream), the most cultured fish of the family *Sparidae* and a model taxon in aquaculture research (3).

*A. fischeri* 5LC was retrieved from gilthead sea bream larvae, 34 days after hatching, on thiosulfate-citrate-bile salts-sucrose agar (TCBS, Oxoid, USA) after 7 days of incubation at 22°C (4). Genomic DNA was extracted from a pure culture grown in marine broth for 2 days at 19°C using the Wizard Genomic DNA purification kit (Promega Corporation, USA). Paired-end sequence reads (125 cycles) were generated using an Illumina HiSeq2500 platform at BaseClear (Leiden, The Netherlands). Sequencing output was 393 Mb, consisting of 2 × 128-bp quality-filtered paired-end reads, leading to genome coverage of ~92×. Sequence reads were assembled into 54 contigs with the “*de novo* assembly” option within the CLC Genomics Workbench (version 7.0.4), and annotation was performed with the RAST (Rapid Annotation using Subsystem Technology) server, version 2.0 (5). *A. fischeri* 5LC shares 99.8% 16S rRNA gene homology with the type strain of the species, *A. fischeri* ATCC 744 (1), and 99.3% and 100% homology with the canonical strains ES114, isolated from *E. scolopes* (6), and MJ11, isolated from the pinecone fish *Monocentris japonica* (7), respectively. The *A. fischeri* 5LC draft genome has 4,250,496 bp and a GC content of 39.0%. Altogether, 3,846 coding sequences (CDSs), 79 tRNAs, and one complete rRNA operon were identified. As usual among the *Vibrionaceae*, the occurrence of the full bacterial luciferase gene cassette *luxCDABE*, along with the autoinducer and transcriptional activator genes *luxI* and *luxR*, is suggestive of a cell density-dependent regulation of bioluminescence in *A. fischeri* 5LC. Conspicuously, all 18 genes of the Syp exopolysaccharide biosynthetic operon, which underpins biofilm formation in *A. fischeri* (8), is present in the 5LC genome. However, in line with observations made for strains MJ11 (7) and SR5—isolated from the squid *Sepiola robusta* (9)—we did not find the regulatory gene *rscS* needed for successful squid colonization by strain ES114 (7). Several other traits encrypted in the 5LC genome serve as hallmarks of a true host-associated lifestyle. These include a vast gene repertoire underlying chemotaxis and flagellar motility, type I, II, III, IV, and VIII protein secretion systems, and bacteriocin biosynthesis potential. Future in-depth mining of the *A. fischeri* 5LC data will contribute to a better understanding of the extent of functional conservation versus plasticity of the species through comparative genomics.

### Nucleotide sequence accession numbers.

The genome sequence of *Aliivibrio fischeri* strain 5LC has been deposited in the DDBJ/ENA-EBI/NCBI databases under the accession number CVOI00000000. The version described in this study is the first version, CVOI01000000, and consists of contig sequences CVOI01000001 to CVOI01000054.
